# Health conditions of inmates in Italy

**DOI:** 10.1186/s12889-016-3830-2

**Published:** 2016-11-16

**Authors:** Fabio Voller, Caterina Silvestri, Gianrocco Martino, Eleonora Fanti, Giorgio Bazzerla, Fabio Ferrari, Marco Grignani, Sandro Libianchi, Antonio Maria Pagano, Franco Scarpa, Cristina Stasi, Teresa Di Fiandra

**Affiliations:** 1 Observatory of Epidemiology, Regional Health Agency of Tuscany, Florence, Italy; 2 Primary Care Unit, District North, Local Social and Health Care Units, Treviso, Italy; 3 Disabled Health Service “Spezzino”, National Healthcare Company, La Spezia, Italy; 4 Department of Mental Health, Healthcare Company of Umbria, Perugia, Italy; 5 Medical House Unit in prison and the third House of Rebibbia, Rome, Italy; 6 Unit for the Overcoming of the Judicial Psychiatric Hospitals and for Mental Health in Prisons, Local Health Salerno, Salerno, Italy; 7 “Health in Prison” Complex Unit, Empoli, Italy; 8 Department of Experimental and Clinical Medicine, University of Florence, Florence, Italy; 9 General Directorate for Health Prevention, Ministry of Health, Rome, Italy

## Abstract

**Background:**

Several studies have shown that prison is characterized by a higher prevalence of chronic diseases than unconfined settings. The aim of this study was to describe the characteristics and health of inmates, focusing on internal diseases.

**Methods:**

We designed a specific clinical record using the Python programming language. We considered all of the diagnoses according to the ICD-9-CM.

**Results:**

Of a total of 17,086 inmates, 15,751 were enrolled in our study (M = 14,835; F = 869), corresponding to 92.2% of the entire inmate population (mean age of 39.6 years). The project involved a total of 57 detention facilities in six Italian regions (for a total of 28% of all detainees in Italy), as counted in a census taken on February 3, 2014. From the entire study sample, 32.5% of prisoners did not present any disorders, while 67.5% suffered from at least one disease. The most frequent pathologies were psychiatric (41.3%), digestive (14.5%), infectious (11.5%), cardiovascular (11.4%), endocrine, metabolic, and immune (8.6%), and respiratory (5.4%).

**Conclusion:**

The findings showed that a large number of detainees were affected by several chronic conditions such as hypertension, dyslipidemia and type 2 diabetes mellitus, with an unusually high prevalence for such a young population. Therefore, a series of preventive measures is recommended to strengthen the entire care process and improve the health and living conditions of prisoners.

## Background

More than 10.2 million people are held in penal institutions throughout the world (as pretrial detainees/remand prisoners or sentenced prisoners). Almost half of these individuals are in the United States, Russia or China, and at least 650,000 people in China and 150,000 people in North Korea are reported to be in pretrial or ‘administrative’ detention. In the past decade, in more than two-thirds of all countries worldwide, the number of people in prisons has been increasing, and this number rose by more than 1 million between the late 1990s and 2013 [1].

In some prisons, individuals suffer from poorer health than the general population and bear a substantial burden of physical and psychiatric disorders [2, 3]. Studies have shown that this situation is common in all high-income countries, where the majority of these surveys were conducted. Health disparities between prisoners and the general population have been linked to various behavioural and socioeconomic factors: high rates of intravenous drug use, which lead to an increased risk of infectious diseases and mental illness; increased alcohol misuse and smoking, which in turn raise the risk of cardiovascular diseases and of some types of cancer [4]. Inmates are affected by social, psychological and physical health problems, such as infectious diseases, chronic viral hepatitis [5], chronic diseases, injuries, dermatological and sensory problems, mental disorders, as well as suicidal and risky behaviours [6–9].

Prison settings involve a high risk of contracting particular diseases [3]. Since detainees are usually released after serving a term of imprisonment, upon which they interact with communities living outside of prisons, they present a complex and difficult challenge for public health, especially with regard to tackling communicable diseases such as HIV or tuberculosis.

While prisoners share the same rights to health and well-being as others, an analysis of European assessments on human rights shows that a combination of poor practices in prisons often exists across Europe. In fact, prisoners’ rights to health are frequently disregarded, and many states insufficiently meet their specific responsibilities of care, causing prisoners to be frequently subjected to avoidable health risks. For example, due to a lack of access to screening or immunization programmes or active case-finding programmes, health personnel often do not act independently from prison authorities but rather are involved in the conflicts between providing healthcare to prisoners and the efforts of authorities to discipline and punish prisoners, such that the public health challenges of prisons are not adequately met [10].

The aforementioned health risks are frequently aggravated by the unhealthy conditions of imprisonment such as lack of space, lack of light and fresh air, lack of clean sanitary facilities or means for personal hygiene, inadequate nutrition and violence [3]. A major aggravating factor that currently occurs in many countries is overcrowding [11].

Furthermore, the Italian National Health Service does not often meet the needs of prisoners due to various factors such as prisoners’ promiscuity, excessive smoking, sedentary life, and stress [12]. Although many surveys have collected data at a regional or local level particularly related to specific health problems such as mental disorders and infectious diseases [13–19], their cross-sectional nature prevents the regular assessment of health-related prevalence and trends. The different healthcare monitoring systems in Italian prisons do not allow for the planning of meaningful interventions unless the extent and types of health problems are known. Therefore, there is interest at the national level to develop a standard monitoring system. The elements currently being discussed are the data to be included in such a system, appropriate system characteristics, and project feasibility.

Due to the lack of descriptions of the prevalence and features of chronic diseases, we hypothesized that the computerized clinical data could facilitate health intervention programmes targeted to prison populations. We speculated that the identification of individuals’ health status in prison could provide useful information in the management of this young and marginalized patient population. Therefore, the aim of this study was to investigate the health status of 17,279 inmates from 6 Italian regions and the prevalence of the main chronic diseases in prisons, focusing on internal diseases, using the 9^th^ revision of the Clinical Modification of the International Classification of Diseases (ICD-9-CM) during a specific project of the Italian Centre for Disease Prevention and Control. We also analysed the treatments associated with these pathological conditions.

## Methods

Due to the lack of any computerized clinical data on inmate populations in every prison involved in this study, we designed a specific clinical record using the Python programming language. The record consisted of two sections: a socio-demographic section and a health section.

The first section included general information such as age, gender, nationality, years of study, as well as the origin of the prisoner from non-detainment, another institute, Diagnostic Therapy Centre (CDT), social security or house arrest. First-time detention was taken into consideration when assessing the health condition of people accessing the penitentiary system.

The second section included the following information: every disease diagnosis (one primary diagnosis and an unlimited number of secondary diagnoses) related to both general medical health and mental health/psychiatric, according to the ICD-9-CM; all pharmacological treatments of prisoners, classified by generic drug and brand name with corresponding daily dose, formulation and route of administration; daily tobacco and cigarette use (former smokers were included among non-smokers); weight and height to calculate BMI; the number of daily hours spent in one’s own cell; specific information about suicide attempts and self-harm including any episodes during the past year of detention, number and method of episodes. Illegal drug use was not evaluated because it was strictly prohibited in the prisons.

The survey was carried out by medical doctors employed or temporarily assigned to the penitentiary for this project. The study was conducted from February 4, 2014, to June 4, 2014, and involved all inmates who were counted in a census conducted at midnight on February 3, 2014, in all prisons involved in the study.

After completing a training to use the computerized tool, the medical doctors filled out clinical records for every prisoner at one time point during the survey administration (point prevalence). Furthermore, the high number and turnover of prisoners in some institutions caused challenges to obtaining a full coverage of the patients during the survey. Moreover, the potential sources of bias included the possibility of undiagnosed diseases and variability among clinicians related to different collections of medical records.

To respect the privacy policy, the software used in the study anonymized the data by converting each prisoner’s personal details into an alphanumeric code. The exported file system (containing the anonymous information) was sent to the Regional Agency of Tuscany via the internet through a secure channel, which was accessed using a login and password on the agency’s website.

The study complied with the Declaration of Helsinki and with the protection of personal data. No identifiable human data were used for this study. In accordance with the Italian law on data confidentiality, the dataset used was not openly available (decree no. 196/2003).

All of the statistical tests were performed at the *p* ≤ 0.05 level (two-sided). Statistical analyses were performed using Stata 12 (College Station, TX, United States). Odds ratios (OR) and 95% confidence intervals (CI) were also calculated to assess associations with demographic and behavioural variables. Independent associations were evaluated by calculating the adjusted OR by multivariate analysis, considering the following variables: age, gender, citizenship, educational qualification, origin of the detainee, duration of imprisonment, smoking, and BMI to control for confounding. The missing data were omitted from the analysis.

## Results

Of the 17,086 inmates, 15,751 (92.2%) were enrolled in our study (M = 14,835; F = 869) (mean age of 39.6 years; 94.2% males and 5.5% females). The inmates were imprisoned in 57 different Italian facilities, with 28% of the total number in national facilities; 53.7% of the participants were Italians, while 46.3% were participants from other EU/non-EU countries; 71% of the prison population were smokers, and the average number of cigarettes smoked per day was 18.6. Of the total population enrolled, 13.1% were obese, 35.2% were overweight, 1.9% were underweight, and 49.7% were normal weight. There were no major differences between inmates’ BMI scores and those of the general population, but there was a higher percentage of obese participants among prisoners (2.8%). The presence of a pathological condition, even if not severe, was found in 67.5% of the total number of inmates enrolled. The total number of diagnoses was 23,031, with an average of 2.2 diagnoses per detainee. A mean of 2.2 diseases was reported for each detainee. The main diseases are listed in Table 1. However, seemingly good health conditions were detected in 32.5% of the total population enrolled.

**Table 1:** Prevalence of diseases in the study population- Italy - 2014

Study population	N	%
Psychiatric disorders	6.504	41.3
Diseases of the digestive system	2.286	14.5
Infectious and parasitic diseases	1.812	11.5
Diseases of the circulatory system	1.788	11.4
Endocrine, nutritional and metabolic diseases, and immunity disorders	1.348	8.6
Diseases of the respiratory system	854	5.4
Diseases of the musculoskeletal system and connective tissue	791	5.0
Symptoms, signs, and ill-defined conditions	809	5.1
Diseases of the nervous system	626	4.0
Diseases of the genitourinary system	452	2.9
Injury and poisoning	344	2.2
Diseases of the skin and subcutaneous tissue	282	1.8
Neoplasms	135	0.9
Congenital anomalies	103	0.7
Diseases of the blood and blood-forming organs	81	0.5
Certain conditions originating in the perinatal period	7	0.0
Complications of pregnancy, childbirth, and the puerperium	4	0.0

The percentage of missing data was 0.03% for age, 0% for gender, 8.6% for citizenship, 35.2% for educational qualification, 6.8% for origin of the detainee, 10% for duration of imprisonment, 16.7% for smoking, and 12.3% for BMI. Data for a complete record (person) were omitted if any information was missing.

### Diseases of the digestive system

Diseases of the oral cavity, salivary glands, and jaws were included in the category of digestive system diseases in accordance with the ICD-9-CM (520–529). Therefore, when considered part of the entire group, these disorders were found to be prevalent (6.2%).

The study also included a subgroup of diseases related exclusively to the gastrointestinal tract (except for chronic viral hepatitis, described by Stasi et al. [5]). Within this subgroup, esophagitis was the most prevalent disorder (6.1%). Using a multivariate analysis, we found significant positive associations with esophagitis for people in the 40–59 age group; people who came from other detention settings (AOR = 1.52), and smokers (AOR = 2.21). We also found significant positive associations for people from Eastern Europe (Table 2).

**Table 2:** Summary statistics, crude odds ratio (COR) and adjusted odds ratio (AOR) for Esophagitis

	Esophagitis		COR	95% CI	AOR	95% CI
	N	%				
Total	977	1.0				
Age (in years)						
18–39	443	5.3	1		1	
40–49	281	6.7	1.30*	1.11–1.52	1.29*	1.06–1.56
50–59	181	8.1	1.59*	1.33–1.90	1.43*	1.13–1.81
60 e +	72	8.1	1.58*	2.22–2.05	1.32	0.92–1.89
Gender						
Male	909	6.1	1		-	-
Female	65	7.5	1.24	0.95–1.61	-	-
Transgender	3	6.4	1.04	0.32–3.37	-	-
Citizenship						
Italian	558	7.2	1.56*	1.26–1.93	1.26	0.96–1.65
Northern Africa	106	4.8	1		1	
Eastern Europe	196	6.8	1.46*	1.14–1.86	1.56*	1.16–2.09
Other Countries	83	5.4	1.14	0.85–1.53	1.36	0.95–1.94
Educational qualification						
Lower middle	658	7.5	1			
Upper intermediate	86	5.9	0.78*	0.62–0.98	0.82	0.64–1.05
Origin of the detainee						
From freedom without detention	304	5.1	1		1	
From freedom with previous detention	647	7.4	1.49*	1.30–1.72	1.52*	1.28–1.79
Duration of imprisonments						
< 1 year	527	6.1	1			
> 1 year	405	7.4	1.24*	1.08–1.41	1.09	0.93–1.29
Smoke						
No	168	4.4	1		1	
Yes	726	7.8	1.82*	1.53–2.16	2.21*	1.77–2,75
BMI						
Normal weight	427	6.2	1		-	-
Underweight	14	5.3	0.85	0.49–1.47	-	-
Obese	133	7.3	1.19	0.98–1.46	-	-
Overweight	345	7.1	1.15	0.99–1.33	-	-

*Abbreviations: COR* crude odds ratio, *AOR* adjusted odds ratio, *95% CI* 95% confidence interval; Crude and adjusted odds ratios (OR) and 95% confidence intervals (variables found to be associated through a univariate analysis were entered into the multivariate model).

*Significant association (*P*<0.05)

### Cardiovascular system

Hypertension was the most prevalent disorder among the group of cardiovascular diseases, although it was differently distributed across the participating regions. When all of the patients were divided by age group (Table 3), the > 40 age group in the cohort of patients with hypertension had the highest prevalence. The multivariate analysis showed significant positive associations between hypertension and people greater than 40 years of age and obese and overweight people (AOR = 4.62 and AOR = 2.20) (Table 3). Italians were at a higher risk of developing hypertension than all other groups (AOR = 1.71).

**Table 3:** Summary statistics, crude odds ratio (COR) and adjusted odds ratio (AOR) for Hypertension

Variable	Hypertension		COR	95% CI	AOR	95% CI
	N	%				
Total	1.112	7.1				
Age (in years)						
18–39	115	1.4	1		1	
40–49	318	7.6	5.95*	4.79–7.39	4.62*	3.46–6.19
50–59	403	18.0	15.87*	12.83–19.65	11.72*	8.74–15.70
60 e +	276	30.9	32.35*	25.64–40.82	22.50*	16.21–31.22
Gender						
Male	1.039	7.0	1		-	-
Female	71	8.2	1.18	0.92–1.52	-	-
Transgender	2	4.3	0.59	0.14–2.44	-	-
Citizenship						
Italian	844	10.9	4.27*	3.63–5.03	1.71*	1.36–2.16
Foreign	186	2.8	1		1	
Educational qualification						
Lower middle	617	7.1	1		1	
Upper intermediate	128	8.8	1.28*	1.05–1.56	1.01	0.80–1.28
Origin of the detainee						
From freedom without detention	342	5.7	1		1	
From freedom with previous detention	719	8.3	1.48*	1.29–1.69	1.18	0.98–1.42
Duration of imprisonments						
< 1 year	544	6.3	1			
> 1 year	487	8.9	1.46*	1.29–1.66	1.09	0.91–1.30
Smoke						
No	351	9.3	1		1	
Yes	593	6.4	0,66*	0.58–0.76	0.86	0.71–1.04
BMI						
Normal weight	226	3.3	1		1	
Underweight	6	2.3	0.69	0.30–1.57	1.40	0.49–3.98
Obese	325	17.9	6.42*	5.37–7.68	4,61*	3.62–5.88
Overweight	445	9.1	2.96*	2.51–3.49	2.20*	1.77–2.72

*Abbreviations: COR* crude odds ratio, *AOR* adjusted odds ratio, *95% CI* 95% confidence interval; Crude and adjusted odds ratios (OR) and 95% confidence intervals (variables found to be associated through a univariate analysis were entered into the multivariate model).

*Significant association (*P*<0.05)

### Endocrine, nutritional and metabolic diseases

Dyslipidemia was the most prevalent disorder (3.7%) among endocrine, nutritional and metabolic diseases, and the second most prevalent was diabetes (3.1%). When all of the patients were divided by age group (Table 4), the > 40 age group in the cohort of patients with endocrine, nutritional and metabolic diseases had the highest prevalence. The multivariate analysis showed significant positive associations between diabetes and people greater than 40 years of age and obese and overweight people (AOR = 3.39 and AOR = 1.56) (Table 4).

**Table 4:** Summary statistics, crude odds ratio (COR) and adjusted odds ratio (AOR) for Diabetes

Variable	Diabetes		COR	95% CI	AOR	95% CI
	N	%				
Total	492	3.1				
Age (in years)						
18–39	44	0.5	1		1	
40–49	128	3.1	6.02*	4.26–8.50	5.92*	3.66–9.59
50–59	177	7.9	16.36*	11.72–22.85	18.56*	11.54–29.85
60 e +	143	16.0	36.35*	25.72–51,38	36.07*	21.66–60.06
Gender						
Male	467	3.2	1		-	-
Female	24	2.8	0.87	0.58–1.32	-	-
Transgender	1	2.1	0.67	0.09–4.86	-	-
Citizenship						
Italian	341	4.4	2.52*	2.04–3.11	0.79	0.58–1.07
Foreign	120	1.8	1		1	
Educational qualification						
Lower middle	277	3.2	1		1	
Upper intermediate	61	4.2	1.35*	1.01–1.79	0.91	0.65–1.27
Origin of the detainee						
From freedom without detention	169	2.8	1		1	
From freedom with previous detention	301	3.5	1.23*	1.01–1.48	1.02	0.79–1.32
Duration of imprisonments						
< 1 year	243	2.8	1			
> 1 year	213	3.9	1.41*	1.17–1.70	1.24	0.97–1.60
Smoke						
No	151	4.0	1		1	
Yes	258	2.8	0.69*	0.56–0.84	0.87	0.66–1.13
BMI						
Normal weight	97	1.4	1		1	
Underweight	1	0.4	0.27	0.04–1.93	0.58	0.08–4.26
Obese	151	8.3	6.34*	4.89–8.23	3.39*	2.44–4.72
Overweight	190	3.9	2.84*	2.21–3.63	1.56*	1.15–2.12

*Abbreviations: COR* crude odds ratio, *AOR* adjusted odds ratio, *95% CI* 95% confidence interval; Crude and adjusted odds ratios (OR) and 95% confidence intervals (variables found to be associated through a univariate analysis were entered into the multivariate model).

*Significant association (*P*<0.05)

### Respiratory system group

Asthma was the most prevalent disorder among the group of respiratory system diseases (Table 5). The multivariate analysis showed significant positive associations between Asthma and people who were female (AOR = 2.46) and obese people (AOR = 1.59). Negative associations were found between respiratory system diseases and people from Eastern Europe and Italy.

**Table 5:** Summary statistics, crude odds ratio (COR) and adjusted odds ratio (AOR) for Asthma

Variable	Asthma		COR	95% CI	AOR	95% CI
	N	%				
Total	271	1.7				
Age (in years)						
18–39	140	1.7	1		1	
40–49	74	1.8	1.07	0.80–1.42	-	-
50–59	41	1.8	1.10	0.78–1.57	-	-
60 e +	16	1.8	1.08	0.64–1.82	-	-
Gender						
Male	237	1.6	1		1	
Female	32	3.7	2.35*	1.62–3.43	2.46*	1.61–3.76
Transgender	2	4.3	2.74	0.66–11.35	2.71	0.63–11.67
Citizenship						
Italian	126	1.6	0.60*	0.44–0,82	0,48*	0.34–0.68
Northern Africa	60	2.7	1		1	
Eastern Europe	27	0.9	0,34*	0.22–0.54	0,31*	0.19–0.49
Other Countries	37	2.4	0.89	0.59–1.34	0.73	0.47–1.14
Educational qualification						
Lower middle	159	1.8	1		-	-
Upper intermediate	35	2.4	1.34	0.92–1.94	-	-
Origin of the detainee						
From freedom without detention	106	1.8	1		-	-
From freedom with previous detention	155	1.8	1.00	0.78–1.28	-	-
Duration of imprisonments						
< 1 year	151	1.7	1		-	-
> 1 year	97	1.8	1.02	0.79–1.32	-	-
Smoke						
No	81	2.1	1		-	-
Yes	153	1.6	0.76	0.58–1.00	-	-
BMI						
Normal weight	109	1.6	1		1	
Underweight	5	1.9	1.21	0.49–2.98	1.05	0.42–2.62
Obese	42	2.3	1.47*	1.03–2.11	1.59*	1.09–2.32
Overweight	87	1.8	1.13	0.85–1.50	1.21	0.89–1.63

*Abbreviations: COR* crude odds ratio, *AOR* adjusted odds ratio, *95% CI* 95% confidence interval; Crude and adjusted odds ratios (OR) and 95% confidence intervals (variables found to be associated through a univariate analysis were entered into the multivariate model).

*Significant association (*P*<0.05)

### Treatments

#### Gastrointestinal diseases

The total number of prescribed drugs associated with the diagnosis of gastrointestinal diseases was 2,043; 62.2% of the prescribed drugs belonged to the gastrointestinal and metabolic system group; 12.9% of the prescribed drugs belonged to the nervous system group; and 7.5% of the prescribed drugs belonged to the musculoskeletal system group.

#### Cardiovascular system

The total number of prescribed drugs associated with cardiocirculatory system diagnoses was 3,552. Of these drugs, 72% acted on the cardiovascular system, and 15.6% acted on blood and blood-forming organs. 6.1% acted on the gastrointestinal tract; 4.7% acted on the nervous system; and 1.6% acted on other bodily systems. Considering only the first two groups of drugs, which acted on the cardiovascular system and blood and blood-forming organs, a strong use of ACE inhibitors not associated with diuretics, such as Ramipril and Enalapril, were found.

#### Endocrine, nutritional and metabolic diseases

The total number of prescribed drugs associated with the diagnosis of endocrine and metabolic diseases was 1873. Of these, 42.4% acted on the gastrointestinal tract, 39.1% acted on the cardiovascular system, 8.4% were used for systemic hormonal preparations (excluding sex hormones and insulin), and 10.1% acted on other bodily systems. In particular, in approximately 65% of the cases of type 2 diabetes mellitus, only oral hypoglycaemic agents were used, essentially represented by biguanides (i.e., metformin); by sulfonylureas, such as glimepiride, glibenclamide, and gliclazide; and by glinides, such as repaglinide; in the remaining 35% of cases, hypoglycaemic agents were associated with short-, rapid- or intermediate-acting insulin.

#### Respiratory system group

The total number of prescribed drugs associated with the diagnosis of respiratory system diseases was 928. Of these, 56.4% acted on the respiratory system; 19.3% were antimicrobial drugs for systemic use; 6.8% acted on the nervous system; 6.4% were used for systemic hormonal preparations (excluding sex hormones and insulin), and 11.2% acted on other bodily systems. In particular, obstructive airway drugs were primarily represented by bronchodilators (approximately 70% of cases) or by aerosol-activated beta2-adrenergic receptors, both long-acting, such as salmeterol, and short-acting, such as salbutamol, in combination with locally acting glucocorticoids such as beclomethasone and fluticasone. Less-prescribed drugs included anticholinergics (12% of cases) such as tiotropium bromide. Even less prescribed were xanthine derivatives, such as theophylline, and leukotriene receptor antagonists, such as montelukast (both in approximately 7% of cases).

## Discussion

First, the study included a high percentage of participants from the target population (92.2% of the total target population). This high percentage was likely due to the fact that clinicians conducted the study on-site. In fact, these clinicians arguably hold positions of power in the study environment. Thus, prisoners likely did not consider opting out of the study as feasible.

Various studies have been conducted on infectious diseases in particular, but to our knowledge, few studies have addressed the general health status of this marginalized population, which in turn can affect the health status of the communities beyond prison. This study was the first in Italy to outline the general health conditions in prison. Therefore, this study could allow preventive actions to be implemented in this setting. In particular, related to hypertension and metabolic disorders, prevention efforts could act on risk factors such as diet, smoking and physical activity. In fact, the results showed that detainees in this study were affected by several chronic conditions, such as hypertension, dyslipidemia and type 2 diabetes mellitus, with an unusually high prevalence for such a young population.

Moreover, a recent systematic review of 21 studies showed a higher prevalence of oral diseases in the prison population than in the general population. In this study, oral diseases were widespread among detainees. This prevalence could have been due to the unavailability of devices for oral hygiene in detention facilities and inmates’ lack of knowledge or care about oral hygiene, resulting in the development of dental caries and periodontal diseases. Some studies [20, 21] suggest the importance of a prison dental service that can provide better access to care and greater awareness of the importance of proper hygiene among prisoners. These programmes have already been implemented, with good results, in England and Wales and the United States. Italian prisons provide dental care services but with a different system. In general, the greater presence of digestive tract diseases among detainees could also be due to styles and habits (high consumption of alcohol and tobacco, use of psychotropic substances, little or no dental hygiene, unbalanced diet, etc.), that inevitably promote the onset of these morbid conditions. Accordingly, this study found that the prevalence of smokers in prison was three times greater than that in the general population (approximately 22%, with an average of approximately 13 cigarettes smoked per day) [22].

Although the prevalence of cardiovascular diseases is lower in prison than in the general Italian population, hypertension, which was diagnosed in 7% of all study participants, was the most common cardiovascular disease among inmates, with a prevalence of 12% in people aged 18 to 65. In the general population, this disease is common among older people. As a result of ageing and a steady increase in the prevalence of associated risk factors, diseases of the circulatory system are the leading cause of death in Italy [23] and across the world [24]. Many of these factors are related to lifestyle, such as smoking, unbalanced diet, physical inactivity, overweight and obesity [25]. Prison is characterized by the presence of most of these factors often also combined with adverse environmental conditions and material and psychological deprivation.

As reported by the National Institutes of Health, related to endocrine, nutritional and metabolic diseases, the prevalence of diabetes in detention facilities is much higher than that of the general population when broken down by age group. In fact, the prevalence of this disease in the general population, among individuals aged 35 to 44 (the age group most represented among prisoners), was only 1.2% [26] compared to 3.1% among prisoners. This finding indicates that diabetes affects more socially disadvantaged and less educated groups, with an increased risk of up to 60% among these groups than those with high levels of education [27]. The potential limitations of this study were possible undiagnosed diseases by clinicians and variability (observer dependent) among clinicians that led to different collections of medical records.

Smoking is one of the most important risk factors of disease within detention facilities because, among detainees, the proportion of smokers is 3 times higher than that observed in the general population [28]. This finding highlights the need to implement measures to reduce the consumption of tobacco in a population that presents strong risk factors for chronic respiratory diseases.

The use of illegal drugs in prison was not examined due to its prohibition. However, evidence suggests that illegal drug use does occur in prisons, likely due to a lack of prison security. Therefore, in this study, the failure to take active illegal drug use into account may present a bias.

## Conclusion

Based on the data collected in the course of this investigation, we must pause and reflect on the health of prison detainees, as the findings that emerged were deeply concerning. First, the lifestyle of the prison population involves various risk factors for the onset of diseases (smoking, alcohol consumption, unbalanced diet). Detainees also become potential bearers of chronic diseases due to factors related to their surroundings, such as cold, damp environments in which they are unable to engage in physical activity. Detainees are often affected by mental disorders, infectious diseases and digestive system diseases. They are also affected by various chronic conditions, such as hypertension, dyslipidemia and type 2 diabetes mellitus, with an unusually high prevalence for such a young population. Therefore, it is necessary to implement a series of preventive measures and strengthen the entire care process to improve the living conditions of prisoners, attempting to eliminate, or at least reduce, risk factors and to adopt care protocols suitable for imprisoned populations. These interventions should include promoting the health of prisoners, the prison staff and the communities outside of prison. The informatization of clinical data would allow health plans to include healthcare resources that are targeted toward prison populations. This system would provide a public health opportunity to screen and treat marginalized groups of patients.

## Abbreviations

CI: Confidence intervals
ICD-9-CM: International Classification of Diseases
OR: Odds ratios

## References

[1] Walmsley R. World prison population list. 10th ed. London: King’s College London International Centre for Prison Studies; 2013. Available at: http://www.prisonstudies.org/sites/default/files/resources/downloads/wppl_10.pdf

[2] Fazel S, Yu R (2011). Psychotic disorders and repeat offending: systematic review and meta-analysis. Schizophr Bull.

[3] Møller L, Stöver H, Jürgens R, et al. Health in prisons: A WHO guide to the essentials in prison health. Geneva: World Health Organization, 2007. Available at: http://www.euro.who.int/__data/assets/pdf_file/0009/99018/E90174.pdf

[4] Fazel S, Gulati G, Linsell L (2009). Schizophrenia and violence: systematic review and meta-analysis. PLoS Med.

[5] Stasi C, Silvestri C, Fanti E, Di Fiandra T, Voller F. Prevalce and features of chronic viral hepatitis and HIV coinfection in Italian prisons. Eur J Intern Med. 2016 (in press). 10.1016/j.ejim.2016.04.020 27189044

[6] Binswanger IA, Krueger PM, Steiner JF (2009). Prevalence of chronic medical conditions among jail and prison inmates in the USA compared with the general population. J Epidemiol Community Health.

[7] Wilper AP, Woolhandler S, Boyd JW (2009). The health and health care of US prisoners: results of a nationwide survey. Am J Public Health.

[8] Shiroma EJ, Ferguson PL, Pickelsimer EE (2010). Prevalence of traumatic brain injury in an offender population: a meta-analysis. J Correct Health Care.

[9] Larney S, Topp L, Indig D (2012). Cross-sectional survey of prevalence and correlates of suicidal ideation and suicide attempts among prisoners in New South Wales, Australia. BMC Public Health.

[10] WHO Regional Office for Europe. Good governance for prison health in the 21st century. A policy brief on the organization of prison health. WHO, 2013.

[11] Aebi MF, Delgrande N (2011). Council of Europe annual penal statistics: SPACE I.

[12] Esposito M (2010). The health of Italian prison inmates today: a critical approach. J Correct Health Care.

[13] Macciò A, Meloni FR, Sisti D (2015). Mental disorders in Italian prisoners: results of the REDiMe study. Psychiatry Res.

[14] Peloso PF, D’Alema M, Fioritti A (2014). Mental health care in prisons and the issue of forensic hospitals in Italy. J Nerv Ment Dis.

[15] Carrà G, Giacobone C, Pozzi F (2004). Prevalence of mental disorder and related treatments in a local jail: a 20-month consecutive case study. Epidemiol Psichiatr Soc.

[16] Brandolini M, Novati S, De Silvestri A (2013). Prevalence and epidemiological correlates and treatment outcome of HCV infection in an Italian prison setting. BMC Public Health.

[17] Sagnelli E, Starnini G, Sagnelli C (2012). Blood born viral infections, sexually transmitted diseases and latent tuberculosis in italian prisons: a preliminary report of a large multicenter study. Eur Rev Med Pharmacol Sci.

[18] Carbonara S, Babudieri S, Longo B (2005). Correlates of Mycobacterium tuberculosis infection in a prison population. Eur Respir J.

[19] Babudieri S, Stamini G, Brunetti B (2003). HIV and related infections in Italian penal institutions: epidemiological and health organization note. Ann Ist Super Sanita.

[20] Heidari E, Bedi R, Makrides NS (2014). Planning for future provision of dental services in prison: an international proposal of two systems. Br Dent J.

[21] Gray R, Fawcett T (2014). Dental triage Hydebank Wood Prison and young offenders centre, Belfast. Br Dent J.

[22] Istituto nazionale di statistica. Indagini Multiscopo “Aspetti della vita quotidiana”. ISTAT 2012. Aivalable at: http://www.istat.it/it/archivio/4630.

[23] Ministero della Salute. Relazione sullo Stato Sanitario del Paese 2012–2013. Available at: http://www.rssp.salute.gov.it/imgs/C_17_pubblicazioni_2258_allegato.pdf.

[24] WHO. The top 10 causes of death. Fact sheet N°310. 2014. Updated May 2014 Available at: http://www.who.int/mediacentre/factsheets/fs310/en/.

[25] Istituto Superiore di Sanità. Il progetto cuore. 2015. Available at: http://www.cuore.iss.it/.

[26] Epicentro. Il portale dell’epidemiologia per la sanità pubblica. La sorveglianza Passi. Il rischio cardivascolare. 2015. Available at: http://www.epicentro.iss.it/passi/dati/cardiovascolare.asp#Ipercolesterolemia.

[27] Epicentro. Il portale dell’epidemiologia per la sanità pubblica. Integrazione, gestione e assistenza. La patologia diabetica: dati di prevalenza a livello nazionale. 2015. Available at: http://www.epicentro.iss.it/igea/diabete/prevalenza.asp.

[28] Parker DR, Fallone D, Martin RA (2014). The relation between smoking status and medical conditions among incarcerated adults. J Addict Med.

